# How Does Career Calling Influence Preservice Teachers' Learning Engagement? A Multiple Mediating Roles of Occupational Self-Efficacy and Vocational Outcome Expectation

**DOI:** 10.3389/fpsyg.2022.874895

**Published:** 2022-05-17

**Authors:** Weiwei Shang, Tianzuo Yu, Xianping Liang, Ji Wang, Jiming Su

**Affiliations:** ^1^School of Marxism, East China Normal University, Shanghai, China; ^2^School of Education, Shanghai Jiao Tong University, Shanghai, China; ^3^Center for Higher Education Research, Southern University of Science and Technology, Shenzhen, China; ^4^School of Humanities, Shandong Agriculture and Engineering University, Jinan, China

**Keywords:** career calling, occupational self-efficacy, learning engagement, social cognitive career theory, preservice teacher, vocational outcome expectation

## Abstract

This study used social cognitive career theory (SCCT) to explore the relationships between career calling, occupational self-efficacy, vocational outcome expectation, and learning engagement among preservice teachers at a normal university in China. Data from 1,029 preservice teachers were analyzed using Structural Equation Modeling. The results revealed that career calling was found to be significantly and positively affected on learning engagement; occupational self-efficacy and vocational outcome expectation were identified as key mediators of this relationship. These findings advance our knowledge of how best to promote the learning engagement of preservice teachers and may inform the future design of teacher development programs.

## Introduction

In the domain of vocational psychology and positive psychology research, career calling (CC) has been a hot topic (Elangovan et al., 2010; Dobrow, 2013; Duffy and Dik, 2013; Zhang et al., 2015), which relates to career development, because of its association with positive individual outcomes, such as work engagement (Xie et al., 2016), satisfaction (Hagmaier and Abele, 2012; Hirschi and Herrmann, 2012; Chen et al., 2016), well-being (Duffy and Dik, 2013), job performance (Park et al., 2015; Kim et al., 2018), and organizational citizenship behavior (Xie et al., 2016). CC can motivate individuals to action, which is related to the deep meaning people feel toward their work (Wrzesniewski, 2003). According to Bryan et al. (2007), CC is a transcendental call that originates from oneself and transcends oneself, and a sense of purpose or meaning and others-oriented values and goals as a source of basic motivation to practice specific life role. CC is important to university student and adult populations, and it can enhance career commitment, career maturity, and work meaning (Duffy and Dik, 2013). Dobrow and Tosti-Kharas (2011) identify the positive effects of calling on career-related efficacy, such as employees who have a strong sense of calling are likely to tend to voluntarily invest more time and energy in it.

Learning engagement is an extension of work engagement in the field of learning, and it is a prerequisite for students to achieve good learning performance and gain professional competence (Chen et al., 2016). Schaufeli et al. (2002) transferred the concept of work engagement to the learning activities of university students, defining it as learning engagement, which is an enduring state of employee fulfillment filled with positive emotions and motivation, including three features of vigor, dedication, and absorption.

In China, the learning engagement of preservice teachers is a critical factor in their professional development, with teacher education occupying an intuitive and important role in the cultivation of teachers. Although teacher education has made remarkable strides in China in recent years, the learning of student teachers remains largely unexplored. However, the available research indicates several issues with the learning of preservice teachers in China: their motivation to learn tends to be weak, with some studies indicating a lack of enthusiasm for learning, different degrees of learning burnout, the serious phenomenon of skipping classes, and so on (Li and Sun, 2011). The study found a strong relationship between learning engagement and positive outcome variables such as students' academic achievement (Howell, 2009). In light of this, the factors influencing academic engagement are gradually coming into the focus of researchers. In Chinese culture, there has always been said that “the teacher is the one who teaches and solves problems” “The engineer of the human soul engineer of the human soul” “the child's cow” “the human ladder” and many other complimentary words of praise has been bestowed on teachers. For the teaching profession, CC is an intuitive factor. However, CC has received limited attention from researchers to date as the factor influencing academic engagement (Duffy and Dik, 2013).

Therefore, the current study aimed to fill this gap by surveying a sample of preservice teachers in China. The study used social cognitive career theory (SCCT; Lent et al., 2002) to investigate the relationship between CC and learning engagement and the mediating effects of occupational self-efficacy and vocational outcome expectation in the relationship between career calling and learning engagement.

## Career Calling and Learning Engagement

CC relates to self-fulfillment, meaningfulness, and happiness in one's career choice and development (Wrzesniewski, 2003). Some special professions, such as teachers, doctors, and other professions whose huge job investment does not match their income, need a sense of career calling even more. Educational work of the teacher is completely devoted to the work that can add to well-being and be meaningful to society. CC is inextricably linked with a high level of work engagement. CC points to the meaning and content of the work that is consistent with one's own inner will, which is essentially autonomous (Elangovan et al., 2010).

Elangovan et al. (2010) believes that CC independently affects levels of work motivation, with its importance exceeding that of other traditional predictors of motivation. Individuals with a strong sense of mission persist under difficult working conditions, even when personal economic interests, time, and energy are sacrificed (Bunderson and Thompson, 2009), and are happy to “live” at work (Dobrow and Tosti-Kharas, 2011). In China, many teachers regard their work as a kind of calling and are devoted to contributing to the growth of children. Since ancient times, teachers in China have taught students how to relate to others, uniting Confucian teachings with their knowledge. If teachers perceive that their profession is important and understand it as a calling, they will focus on inner satisfaction, self-worth, and the meaningfulness it brings to their lives (Zhang, 2012).

The career calling of student teachers contributes to developing a sense of their meaning and value in life (Zhang et al., 2013), influencing motivation, adaptability, and commitment learning (Chen et al., 2016; Cui, 2021). Analyzing results from a sample of postsecondary students from Canada and the United States, Woitowicz (2009) found that a calling was related to intrinsic and extrinsic aspects of academic motivation. However, there is no direct evidence to suggest that a career calling leads to greater engagement in learning, particularly for students in the Chinese educational context. Therefore, based on the evidence above, the first hypothesis of this study was proposed:

Hypothesis 1: Career calling has a positive effect on learning engagement.

## The Mediating Roles of Self Efficacy and Outcome Expectation

In addition to the direct effect, after the sense of career calling triggers work motivation, it may also indirectly affect work engagement by affecting other work psychology and behavior and work results. Some studies detected the mediating effect of professional identity and self-efficacy (Hirchi, 2012). Xie et al. examined the mediating role of career adaptability between sense of purpose and job engagement, and career satisfaction (Xie et al., 2016). Social cognitive career theory (SCCT) applies Bandura's social cognitive theory (e.g., Bandura, 1977) to the professional field. SCCT seeks to trace the web of connections between people and their careers, focusing specifically on cognitive and contextual factors (Lent et al., 2002). An individual's capacity to control his/her own cognition, motivation, affect, and action operates through mechanisms of personal agency (Bandura, 1989). SCCT is a motivational theory driven by self-efficacy, outcome expectations, and goal-directed activity (Kassean et al., 2015), which has been widely accepted and is an empirically validated model (Brown et al., 2011; Chin and Rasdi, 2014; Duffy et al., 2014a,b) enabling changes behavioral and cognitive changes to be predicted. The theory claims that behavior is influenced by both cognitive and environmental factors.

In the literature, two distinct conceptualizations of self-efficacy are prevalent: domain-specific self-efficacy (Arenius and Minniti, 2005; Zhao et al., 2005) and generalized self-efficacy (Baum et al., 2001; Markman et al., 2005). SCCT presumes domain-specific self-efficacy to consist of a continuously evolving set of self-beliefs that are in constant interaction with other person inputs, environmental inputs, and behavioral factors. These beliefs develop through four mechanisms: mastery experiences, vicarious experiences, social persuasion, and physiological factors (Wood and Bandura, 1989). Occupational Self-efficacy is the key structure of SCCT, which refers to the belief of employees in their own workplace success (Brown et al., 2011; Chang and Edwards, 2015), and is believed to directly impact behavior (Brown et al., 2011; Duffy et al., 2014a; Chang and Edwards, 2015; Liguori et al., 2019). It is widely held to inspire spontaneous engagement in work (Caesens and Stinglhamber, 2014). Students with high self-efficacy are more willing to spend extra energy and time to complete learning tasks and concentrate more on school-related activities (Siu et al., 2014), resulting in more learning engagement (Martin and Rimm-Kaufman, 2015).

Many studies have examined core cognitive variables in SCCT and their relationship with various personal and environmental factors (Chin and Rasdi, 2014; Chang and Edwards, 2015). However, this research has sidelined the importance of learning experience, which is closely related to personal-environmental factors in SCCT. The theoretical importance of learning experience is fully reflected in SCCT, and earlier scholars highlighted the urgent need for further studies (Schaub and Tokar, 2005; Tokar et al., 2012). In SCCT, the learning experience is viewed as an important component of occupational self-efficacy (Lent et al., 1994). Within the framework of the SCCT theory, CC is a learning experience (Domene, 2012). Students in universities with more developed senses of CC often show a higher degree of professional self-efficacy (Dik and Steger, 2008; Domene, 2012; Kaminsky and Behrend, 2015). When individuals have a sense of purpose for occupation, they will highly recognize it and consider their occupation to be important and meaningful, so they will actively engage in related occupational activities (Dobrow and Tosti-Kharas, 2011; Hirschi and Herrmann, 2012). Similarly, Sellers and colleagues found that Christian women's sense of being called to their work was an important force in maintaining their ongoing involvement and passion for their careers (Sellers et al., 2005). The university level is the stage of career preparation for preservice teachers. To acquire the vocational competencies to achieve their sense of purpose, they may actively engage in relevant career preparation activities. Thus, the second hypothesis of this research was as follows:

Hypothesis 2: Occupational self-efficacy mediates the relationship between career calling and learning engagement.

The second component of SCCT is vocational outcome expectation (VOE), which represents a person's judgment of the consequences of the execution or non-execution of a specific behavior (Dik and Steger, 2008; Brown et al., 2011; Domene, 2012; Caesens and Stinglhamber, 2014; Chin and Rasdi, 2014; Kaminsky and Behrend, 2015). Outcome expectations are beliefs about the consequences of behavior (Lent and Brown, 2008), including beliefs about the outcomes assumed to result from the behavior itself (Lent et al., 2002). Woitowicz and Domene (2011) demonstrated that a calling was a strong, positive predictor of vocational outcome expectation. Similarly, Dik and Steger found a significant correlation between vocational outcome expectation and the presence of CC in undergraduate students from the United States (Dik and Steger, 2008). Within SCCT, career calling as learning experiences influence learning engagement directly and also indirectly, through the mediating variable of vocational outcome expectation. Yet the relative strength of the direct and indirect relations between calling and learning engagement remains unclear. Thus, the following hypothesis is proposed:

Hypothesis 3: Vocational outcome expectation mediates the relationship between career calling and learning engagement.

In summary, occupational self-efficacy and vocational outcome expectation are important links that connect career calling and learning engagement. SCCT theory particularly emphasizes the influence of self-efficacy and outcome expectation on people's career performance (Dik and Steger, 2008; Duffy et al., 2011). Outcome expectation beliefs result from learning experiences and may be influenced by self-efficacy beliefs when outcomes depend on the quality of one's ability. Analyzing a sample of 855 undergraduate students from three universities in Atlantic Canada, Domene (2012) suggested that the relation between a sense of calling and expectations of successful future occupational outcomes was predominantly indirect, working through self-efficacy. Moreover, teachers' sense of a calling had significantly positive predictive effects on job performance. Given that learning engagement represents the extension of work engagement into the field of learning, and is an important indicator of personal behavior (Schaufeli et al., 2002; Salanova et al., 2010; Christian et al., 2011), it is reasonable to assume that preservice students with a career calling may express greater self-efficacy and higher expectations of successful vocational outcomes in the future, thereby enhancing their learning engagement at university. This is expressed in our fourth and final hypothesis.

Hypothesis 4: Career calling can affect learning engagement through the serial mediating roles of occupational self-efficacy and vocational outcome expectation.

**Figure 1:**
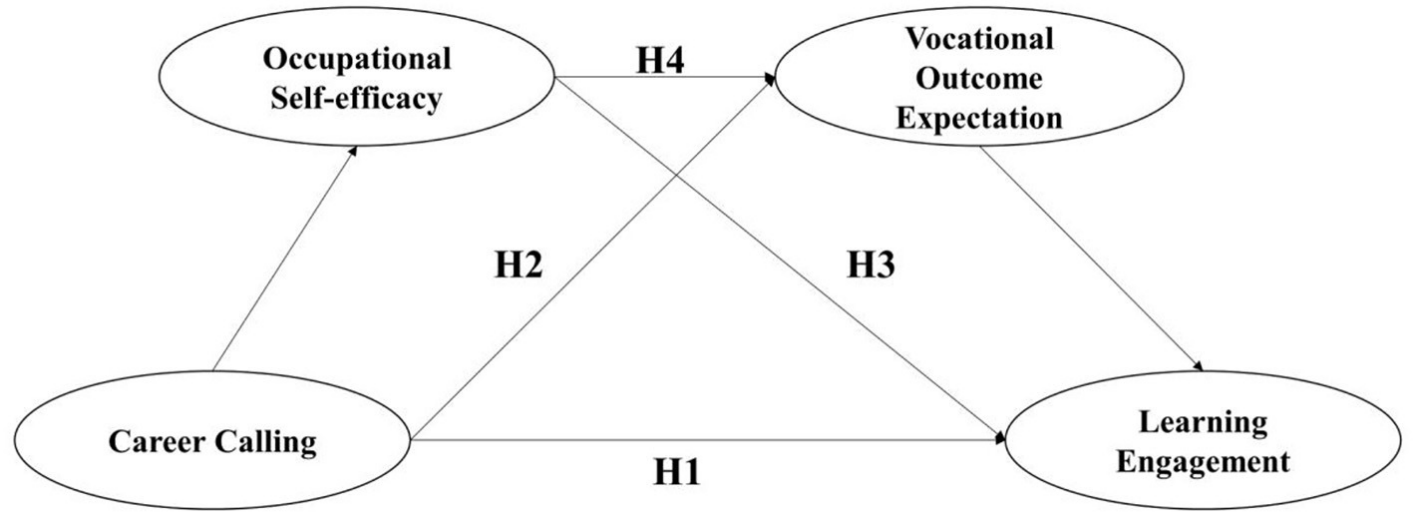
Hypothesized model of mediated relationships between career calling and learning engagement.

Hypotheses 1–4 are exploratory and reflect the fact that research on preservice teachers' learning engagement in China is still at an early stage. We used the SCCT framework to further understand the impact of career callings on the learning engagement of preservice teachers at a normal university in China. More specifically, this study aimed to examine the internal mechanism by which career calling affects learning engagement through the mediating roles of occupational self-efficacy and vocational outcome expectation. In doing so, it aimed to provide a solid theoretical rationale for improving preservice teachers' engagement in learning and the quality of teacher education they receive. Figure 1 depicts the research model.

## Materials and Methods

### Participants and Procedure

Between February and March 2020, the COVID-19 pandemic in China limited our collection of data to an online questionnaire survey platform (Wen Juanxing). Participants were notified about the survey via Wechat and email. To reduce dropout rates, short questionnaires were used to measure the research variables. The questionnaire could be submitted only after all the items had been completed in order to maximize valid return rates. The participants completed the questionnaires anonymously and were informed that all responses would remain confidential, with data securely stored, only accessible to the research team, and only used for research purposes. The present study obtained informed consent to the study from all participants and approval from the ethical committee of East China Normal University.

The participants were recruited from East China Normal University (Shanghai), a leading center of teacher education in China that features in the central government's Double First Class University Plan. A convenience sampling strategy underpinned the web-based distribution of the questionnaires, which 1,078 students voluntarily completed. After responses with duplicate or contradictory responses had been removed, a total of 1,029 valid responses remained, a rate of 95.45%. The participants comprised 280 males (27.2%) and 749 females (72.8%). There were 237 freshmen (23%), 325 sophomores (31.6%), 316 juniors (30.7%), and 151 seniors (14.7%). Among these, 652 students (63.4%) were from rural backgrounds, and 377 (36.6%) were from cities.

### Measures

#### Learning Engagement Scale

Learning engagement was assessed using the Chinese version of the Utrecht Work Engagement Scale for Students (UWES-S; Schaufeli et al., 2002; Gan et al., 2007). The UWES-S measures three factors: vigor (six items), dedication (five items), and absorption (six items). Participant responses are recorded on a 5-point scale from “never” (1) to “every day” (5), with higher scores representing higher levels of engagement. The Cronbach's alpha coefficient in the present study was 0.94.

#### Career Calling Scale

Career Calling was assessed using the Brief Calling Scale (Dobrow and Tosti-Kharas, 2011), whose reliability and validity was established by its originators and subsequently confirmed (Lv et al., 2021). The scale consists of 12 items (e.g., “I would sacrifice everything to be a teacher” or “I would continue being a teacher even in the face of severe obstacles”). Items were rated on a 5-point Likert scale (1 = strongly disagree and 5 = strongly agree). Higher mean scores show a stronger career calling; the Cronbach's alpha coefficient for this scale in the present study was 0.95.

#### Occupational Self-Efficacy Scale

Occupational self-efficacy was assessed using the short form version from Rigotti et al. (2008). This scale consisted of six items (e.g., “I feel prepared for most of the demands of my job” or “I can remain calm when facing difficulties in my job because I can rely on my abilities.” Items were rated on a 5-point Likert scale (1 = not true at all and 5 = mostly true), with higher scores representing greater levels of occupational self-efficacy. The Cronbach's alpha coefficient for this scale in the present study was 0.92.

#### Vocational Outcome Expectation Scale

The VOE scale (McWhirter et al., 2000) was used to assess the extent to which participants had the sense of being able to achieve a successful vocational outcome grounded in SCCT. The scale consists of six items that ask participants to record their sense of being able to achieve a successful vocational outcome (e.g., “I will be successful in my chosen occupation” or “My career planning will lead to a satisfying career for me”). The items were rated on a 5-point Likert scale (1 = strongly disagree and 5 = strongly agree), with higher scores reflecting better vocational outcome expectations. The scale has previously been used to examine VOE among international students attending an American university (Reynolds and Constantine, 2007). The Cronbach's alpha coefficient for this scale in the present study was 0.93.

### Data Analysis

The major goal of the present study is to examine a moderated mediation hypothesis. Data were analyzed with SPSS Version 25.0. Mplus 8 was adopted to perform the multiple mediation model. First, a missing value analysis was carried out to examine patterns in the missing responses. The result showed that missing values were <5% for every variable; thus, an expectation-maximization algorithm was used to handle missing data in the analysis. Second, descriptive statistics (M, SD, skewness, and kurtosis) and correlations were calculated. Cronbach's α coefficients were used to examine the subscales' reliability. Third, we used confirmatory factor analysis (CFA) to detect the validity of the three constructs, including between-item relationships, latent variables, and fit indices. Fourth, we used structural equation modeling to examine the direct and mediated effects between the variables. Then, we used 5,000 bootstrap samples and the 95% bias-corrected confidence interval (95% CI) to examine the significance of the multiple mediation effect (Hayes, 2013). The statistical significance level was set at p < 0.05.

## Results

### Common-Method Bias Test

To control for common method bias, Harman's single-factor test (Eby and Dobbins, 1997) and the method-factor approach (Xiong et al., 2012) were used. Among the factors, eight had eigenvalues greater than one, with the first factor explaining 22.52% of the total variance, well below the recommended threshold of 40%. Furthermore, the confirmatory factor analysis (CFA) conducted via the method-factor approach showed that the model did not fit the data closely (X^2^/df = 19.142, CFI = 0.491, IFI = 0.914, RMSEA = 0.051). These investigations confirmed the absence of serious common method bias in the data.

### Correlations Between Primary Variables

The Spearman's correlations for the means and SDs of all study measures are presented in Table 1. As the table shows, career calling was positively correlated with occupational self-efficacy (*r* = 0.466, *p* < 0.01), vocational outcome expectation (*r* = 0.469, *p* < 0.01), and learning engagement (*r* = 0.520, *p* < 0.01). Moreover, both occupational self-efficacy and vocational outcome expectation were positively related to learning engagement, with values ranging from moderate to large (0.576 to 0.613).

**Table 1:** Means, standard deviations, correlations, and reliabilities.

**Measures**	**M**	**SD**	**1**	**2**	**3**	**4**
1.CC	3.82	0.757	1.000			
2.OEE	3.79	0.669	0.466^**^	1.000		
3.VOE	4.02	0.674	0.469^**^	0.553^**^	1.000	
4.LE	3.95	0.740	0.520^**^	0.576^**^	0.613^**^	1.000

** *p < 0.01; N = 1,029 preservice teachers; CC, career calling; OEE, occupational self-efficacy; VOE, vocational outcome expectation; LE, learning engagement*.

### Structural Equation Modeling (SEM)

Based on the conceptual model shown in Figure 1, structural equation modeling (SEM) was carried out using AMOS software to examine the relationship between the latent variables of career calling, occupational self-efficacy, vocational outcome expectation, and learning engagement. We controlled for gender and grade by connecting them to the endogenous variables and proceeding to run a series of path analyses. The indices used (Hu and Bentler, 1998; Byrne, 2016) indicated that the model provided a satisfactory fit to the data: X^2^/df = 2.81; comparative fit index (CFI) = 0.988; Tucker Lewis index (TLI) = 0.976; root mean square error of approximation (RMSEA) = 0.059; standardized root means square residual (SRMR) = 0.017.

**Figure 2:**
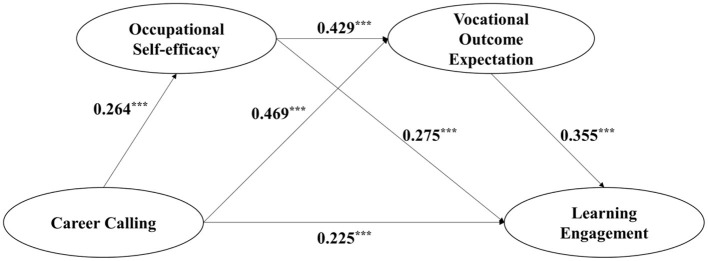
The serial mediation model with occupational self-efficacy and vocational outcome expectation as mediators of the linkage between career calling and learning engagement. ***p <0.001.

Figure 2 shows that all proposed paths in the model were significant at the 0.05 level or above. Career calling had a significant positive effect on occupational self-efficacy (β = 0.264, *p* < 0.001), vocational outcome expectation (β = 0.469, *p* < 0.001), and learning engagement (β = 0.22, *p* < 0.001). Both occupational self-efficacy (β = 0.275, *t* = 4.548, *p* < 0.001) and vocational outcome expectation (β = 0.355, *p* < 0.001) carried a significant positive effect on learning engagement. Moreover, occupational self-efficacy exerted a significant positive effect on vocational outcome expectation (β = 0.429, *p* < 0.001).

### Mediational Roles of Career Calling and Learning Engagement

The bootstrapping method with a 5,000 bootstrap sample was used to test the indirect effects of career calling on learning engagement. Table 2 shows the results after controlling for age (*p* = 0.96) and gender (*p* = 0.31). First, the mediating effect of occupational self-efficacy was 0.073, with 95% CI [0.106, 0.252], excluding 0, thereby supporting hypothesis 2. Second, the mediating effect of vocational outcome expectation was 0.166, with 95% CI [0.031, 0.092], excluding 0, meaning that hypothesis 3 was confirmed. Notably, the size of the mediating effect of occupational self-efficacy was smaller than that of vocational outcome expectation. Finally, we tested the chain multiple mediation effect of these variables. The chain intermediary effect of occupational self-efficacy and vocational outcome expectation (β = 0.040, 95% CI [0.021, 0.062]) was significant, thus providing support for hypothesis 4.

**Table 2:** Bootstrap analyses of significance of mediation (controlling for gender and age).

**Model pathways**	**Effect**	**95% confidence interval**		**Percentage**
		**Boot LLCI**	**Boot ULCI**	
CC → OEE → LE	0.073^***^	0.106	0.252	14.5%
CC → VOE → LE	0.166^***^	0.031	0.092	32.9%
CC → OEE → VOE → LE	0.040^***^	0.021	0.062	7.9%

*** *p <0.001; N = 1,029 preservice teachers; CC, career calling; OEE, occupational self-efficacy; VOE, vocational outcome expectation; LE, learning engagement*.

Overall, these results supported our conceptual model and showed that career calling could both have significant direct effects on learning engagement and indirect ones via the mediation of occupational self-efficacy and vocational outcome expectation.

## Discussion

The question of “what makes a good teacher?” has become the focus of global education reform, particularly teacher education reform. Some scholars have gradually shifted from a focus on the external professional skills of teachers to an exploration of their inner selves and their professionalism. The study of the career calling of preservice teachers is of great value to the study of the cultivation of teacher professionalism and the enrichment of theories of teacher professional development. The present study aimed to examine the direct relationship between career calling and learning engagement and the mediation of this relationship by occupational self-efficacy and vocational outcome expectation among preservice teachers in China. Using structural modeling, we demonstrated that career calling directly predicted learning engagement and that the relationship was mediated by occupational self-efficacy and vocational outcome expectation. Finally, the serial two mediator model indicated that career calling influenced learning engagement via occupational self-efficacy and vocational outcome expectation sequentially.

The findings showed that career calling was significantly related to learning engagement, which supported H1 and supported Duffy and Dik's findings (Duffy and Dik, 2013) by confirming that preservice teachers with a high sense of career calling are more engaged in studying to improve their academic performance. Moreover, the current study extends the scope of previous research on work-based CC to the field of education (Bunderson and Thompson, 2009; Elangovan et al., 2010), indicating the importance of cultivating career calling among preservice teachers at university. The study also enriches empirical research on the impact of CC on the individual career growth and development of preservice students. While previous research focused on the direct effect of career calling on learning engagement (Hall and Chandler, 2005; Duffy et al., 2011, 2012, 2014b; Hirschi and Herrmann, 2012), our study explored this relationship in greater detail by considering the mediating roles of occupational self-efficacy and vocational outcome expectation.

By verifying hypotheses 2 and 3, we have demonstrated that occupational self-efficacy and vocational outcome expectation fulfill important intermediary roles in the relationship between career calling and learning engagement among preservice teachers in China. This supports previous studies such as that of Hirschi and Herrmann (2012), who detected a moderate, positive correlation between career calling and career preparation. The framework of social learning theory (Bandura, 1977) indicates that the learning experience of career calling would contribute to enhancing self-efficacy, in turn improving engagement in learning (Chen et al., 2016). Similarly, SCCT (Brown et al., 2011), holds that occupational self-efficacy and vocational outcome expectation can predict aspects of career performance such as engagement in career preparation. Furthermore, occupational self-efficacy and vocational outcome expectation play a key mediating role in the research model of SCCT.

Our research found that occupational self-efficacy enhanced the level of vocational outcome expectation and significantly mediated the impact of career calling on the learning engagement of Chinese preservice teachers, thereby confirming hypothesis 4 in a manner consistent with the results of previous research. If students can maintain motivation in difficult conditions (Bunderson and Thompson, 2009), their work attitudes will improve (Steger et al., 2010), leading to more positive self-evaluation and further enhancing their sense of occupational self-efficacy. SCCT indicates that, as a form of the learning experience, career calling influences outcome expectation directly, as well as indirectly through the mediating variable of self-efficacy (Domene, 2012). This study supports the predictions of SCCT and demonstrates career calling, occupational self-efficacy, and vocational outcome expectation as prominent factors in the learning engagement of preservice teachers. This is consistent with recent work by Sheu and Bordon (2017) showing that contextual supports have received more attention in international SCCT research. The geographic distribution of international SCCT research showed that more empirical attention is still needed in other countries.

### Limitations and Future Research

As with all research, several limitations should be acknowledged. First, our sample consisted only of trainee teachers in universities rather than those in primary and secondary schools. Extending our sample to include these teachers would allow us to incorporate additional variables relevant to these and other groups, creating a more comprehensive model. Second, the cross-sectional nature of the study excludes causal explanations of the relationships that may exist among career calling, occupational self-efficacy, vocational outcome expectation, and learning engagement. Future studies should consider conducting experimental, prospective, and longitudinal approaches to examine causality among these variables. Third, our focus on the mediating mechanisms of career calling and learning engagement excluded other factors that influence preservice teachers' career calling and learning engagement, such as resilience (Blackwell et al., 2007) and goal achievement orientation (Abrami and McWhaw, 2001). Subsequent research must include these factors as mediating or moderating variables to better understand the relationship between career calling and learning engagement. Forth, This study only analyses the effect of preservice teachers as a whole on their CC, and future analysis of categories or differences needs to be considered, such as grade level, gender, and so on. Finally, the participants in the present study were recruited from mainland China. The extent to which results from the Chinese educational context are replicated in other countries and cultures is a matter for further empirical research. Furthermore, we will conduct field research or other sociological investigations and perform a longitudinal analysis to examine changes in CC and learning engagement by following preservice teachers from their first year through several years after they are hired.

## Conclusion

In conclusion, the present study aimed to explore the direct and indirect predictive factors involved in the learning engagement of preservice teachers in mainland China. It provides an important preliminary understanding of how preservice teachers' career calling influences their learning engagement via occupational self-efficacy and vocational outcome expectation. The study complements existing research on the effect of career calling on learning engagement, using SCCT to introduce occupational self-efficacy and vocational outcome expectation as mediators of the link between career calling and learning engagement. The positive relationship between career calling and learning engagement indicates that university-based teacher trainers should aim to cultivate career calling in their students. Additionally, the powerful mediating effects of occupational self-efficacy and vocational outcome expectation on learning engagement suggest that nurturing the professional self-confidence and commitment of preservice teachers will maximize active engagement in their studies.

## Data Availability Statement

The raw data supporting the conclusions of this article will be made available by the authors, without undue reservation.

## Ethics Statement

The studies involving human participants were reviewed and approved by East China Normal University. The patients/participants provided their written informed consent to participate in this study.

## Author Contributions

All authors listed have made a substantial, direct, and intellectual contribution to the work, and approved it for publication.

## Funding

This study was supported by The National Social Science Fund of Chinese government [No. 21CKS007].

## Conflict of Interest

The authors declare that the research was conducted in the absence of any commercial or financial relationships that could be construed as a potential conflict of interest.

## Publisher's Note

All claims expressed in this article are solely those of the authors and do not necessarily represent those of their affiliated organizations, or those of the publisher, the editors and the reviewers. Any product that may be evaluated in this article, or claim that may be made by its manufacturer, is not guaranteed or endorsed by the publisher.
