# Supplementary material for: How Does Career Calling Influence Preservice Teachers' Learning Engagement? A Multiple Mediating Roles of Occupational Self-Efficacy and Vocational Outcome Expectation
Source: Front Psychol. 2022 May 17;13:874895. doi: 10.3389/fpsyg.2022.874895 (PMC9152323; doi:10.3389/fpsyg.2022.874895)
Supplement: Supplementary file 1 [file Table_1.docx]

**APPENDIX**

**Career Calling Scale Items**

(1)I am passionate about being a teacher.

(2)I enjoy being a teacher more than anything else.

(3)Being a teacher gives me immense personal satisfaction. (4)I would sacrifice everything to be a teacher.

(5)The first thing I often think about when I describe myself to others is that I’m a teacher.

(6)I would continue being a teacher even in the face of severe obstacles.

(7)I know that being a teacher—either professionally or as an amateur will always be part of my life.

(8)I feel a sense of destiny about being a teacher—either amateur or professional.

(9)Teaching is always in my mind in some way.

(10)Even when not doing educational activities, I often think about being a teacher.

(11)My existence would be much less meaningful without my involvement in education

(12)Being a teacher is a deeply moving and gratifying experience for me.

**Learning Engagement Scale Items**

1. At my work, I feel bursting with energy.

2. I find the work that I do full of meaning and purpose.

3. Time flies when I am working.

4. At my job, I feel strong and vigorous.

5. I am enthusiastic about my job.

6. When I am working, I forget everything else around me.

7. My job inspires me.

8. When I get up in the morning, I feel like going to work.

9. I feel happy when I am working intensely.

10. I am proud of the work that I do.

11. I am immersed in my work.

12. I can continue working for very long periods at a time.

13. To me, my job is challenging.

14. I get carried away when I am working.

15. At my job, I am very resilient, mentally.

16. It is difficult to detach myself from my job.

17. At my work, I always persevere, even when things do not go well.

**Occupational Self-efficacy Scale**

1.I can remain calm when facing difficulties in my job because I can rely on my abilities.

2.When I am confronted with a problem in my job, I can usually find several solutions.

3.Whatever comes my way in my job, I can usually handle it.

4.My past experiences in my job have prepared me well for my occupational future.

5.I meet the goals that I set for myself in my job.

6.I feel prepared for most of the demands in my job.

**Vocational Outcome Expectation Scale**

1.My career plan will lead to a satisfying my career.

2.I will be successful in my chosen career/occupation.

3.I will be successful in my chosen career/occupation.

4.My talents and skills will be used in my career/occupation.

5.I will have a career/occupation that is respected in our society.

6.I will achieve my career/vocational goals.
